# Chemokine‐Capturing Wound Contact Layer Rescues Dermal Healing

**DOI:** 10.1002/advs.202100293

**Published:** 2021-07-18

**Authors:** Lucas Schirmer, Passant Atallah, Uwe Freudenberg, Carsten Werner

**Affiliations:** ^1^ Leibniz‐Institut für Polymerforschung Dresden e.V. Hohe Str. 6 Dresden 01069 Germany; ^2^ Technische Universität Dresden Center for Regenerative Therapies Dresden Fetscherstr. 105 Dresden 01307 Germany

**Keywords:** chronic wounds, glycosaminoglycans, hydrogels, wound healing

## Abstract

Excessive inflammation often impedes the healing of chronic wounds. Scavenging of chemokines by multiarmed poly(ethylene glycol)‐glycosaminoglycan (starPEG‐GAG) hydrogels has recently been shown to support regeneration in a diabetic mouse chronic skin wound model. Herein, a textile‐starPEG‐GAG composite wound contact layer (WCL) capable of selectively sequestering pro‐inflammatory chemokines is reported. Systematic variation of the local and integral charge densities of the starPEG‐GAG hydrogel component allows for tailoring its affinity profile for biomolecular signals of the wound milieu. The composite WCL is subsequently tested in a large animal (porcine) model of human wound healing disorders. Dampening excessive inflammatory signals without affecting the levels of pro‐regenerative growth factors, the starPEG‐GAG hydrogel‐based WCL treatment induced healing with increased granulation tissue formation, angiogenesis, and deposition of connective tissue (collagen fibers). Thus, this biomaterials technology expands the scope of a new anti‐inflammatory therapy toward clinical use.

## Graphical abstract

Excessive inflammation impeding the healing of chronic wounds can be rescued through selective scavenging of chemokines by multiarmed poly(ethylene glycol)‐glycosaminoglycan (starPEG‐GAG) hydrogels. Herein, a textile‐starPEG‐GAG composite wound contact layer capable of dampening the excessive inflammatory signals without affecting the levels of pro‐regenerative growth factors is reported and tested in a large animal (porcine) model of human wound healing disorders.

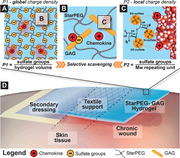

## 1 Introduction

Chronic, non‐healing skin wounds are a major healthcare challenge, becoming increasingly important with rising rates of diabetes and obesity.^[^ ^1^ ^]^ The absence of healing results from a dysregulation of molecular signals that control the immune response and tissue repair. While an initial inflammatory stage is vital in physiological wound healing to fend off infections and clear the damaged tissue, its temporal expansion with excessive infiltration by neutrophils and macrophages^[^ ^2^, ^3^, ^4^ ^]^ producing inflammatory cytokines and chemokines—which in turn attract even more immune cells—can result in a vicious circle of sustained inflammation.^[^ ^5^, ^6^ ^]^ Widely used dressings that protect the breached skin and regulate moisture cannot cure chronically inflamed wounds in which amplified protease levels cause a depletion of pro‐regenerative growth factors and foster fibroblast senescence.^[^ ^6^, ^7^, ^8^, ^9^ ^]^

Chemokines, soluble signaling molecules that trigger the tissue invasion of pro‐inflammatory cells, have been identified as promising targets in the treatment of chronic wounds.^[^ ^10^, ^11^ ^]^ Since chemokine distribution in tissues is controlled by their complexation with sulfated glycosaminoglycans (GAG) of the extracellular matrix (ECM) or cell surface proteoglycans (PG),^[^ ^12^ ^]^ GAG‐based biomaterial technologies offer a means of modulating chemokine levels to dampen excessive inflammation.^[^ ^13^, ^14^ ^]^ Exploring this option, we have previously demonstrated that biohybrid multiarmed poly(ethylene glycol)‐GAG (starPEG‐GAG) hydrogels (containing particular heparin derivatives as GAG component) can act as a “molecular sink” for chemokines, resolve inflammation and enable wound closure in a murine model of delayed wound healing.^[^ ^15^ ^]^

To translate this approach toward clinical applicability, we now designed scalable composite textile wound contact layer (WCL) dressings containing a biostable starPEG‐GAG hydrogel of customized molecular affinity. Particular attention was paid to the suitability of the novel textile composite dressing to sequester inflammatory chemokines without depleting pro‐regenerative factors, its compatibility with effective wound exudate management, and the material's stability under mechanical forces. Maximized sequestration of pro‐inflammatory chemokines of the CC and CXC family was achieved by adjusting the local and integral charge densities of the starPEG‐GAG‐based hydrogel within the WCL according to a biologically inspired rational design concept.

Applying the developed starPEG‐GAG‐hydrogel based WCL composite dressings in the treatment of chronic non‐healing porcine cutaneous wounds, a model with high concordance to human pathophysiology,^[^ ^16^, ^17^, ^18^, ^19^ ^]^ we demonstrated the efficacy and safety of the proposed therapeutic technology. As a key benefit, the approach targets pro‐inflammatory mediators without the administration of drugs or bioactives, offering the ease of use of a conventional biostable WCL that can be combined with various established secondary dressings.

## 2 Results and Discussion

### 2.1 Biologically Inspired Materials Design for Maximized Chemokine Scavenging

**Figure 1:**
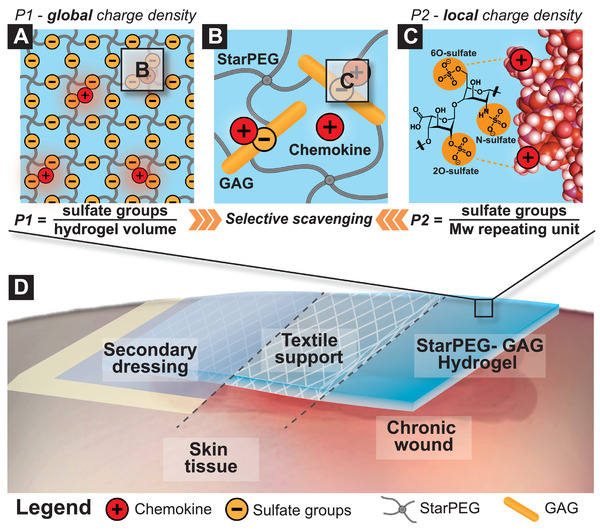
The binding affinity of the hydrogel polymer network for pro‐inflammatory chemokines can be described by two parameters P1 and P2: The A) global charge density P1 that reflects the electrostatic interactions between negatively charged glycosaminoglycan (GAG) building blocks and B) positively charged patches at the chemokine surface and C) the local charge density P2 that is related to a spatially matching attraction between particular charged sulfate moieties at the GAG and single positively charged amino acid residues of the protein surface. The D) textile‐based starPEG‐GAG hydrogel‐based WCL composite dressing is placed on top of the chronic wound where it acts as an “affinity‐based sink” for excessive inflammatory mediators.

The anti‐inflammatory functionality of the developed starPEG‐GAG‐hydrogel based WCL composite dressings relies on the effective complexation of highly anionic sulfated GAGs (heparin and heparin derivatives) with partially positively charged chemokines^[^ ^20^, ^21^ ^]^ (see **Figure** **1**). GAG‐chemokine binding governs the spatial signal distribution in living tissues, including the formation of concentration gradients serving as powerful migratory signals. In these interactions, the localization of the relatively small chemokines (*M*_w_: 8–9 kDa) is primarily controlled by 3D arrangements of sulfated GAGs, defined by the protein core of a PG or the network density of the ECM, that direct interactions with proteins by 1) non‐specific, long‐ranging electrostatics and 2) specific binding by molecular binding motifs involving accumulated positively charged amino acid residues.^[^ ^14^, ^22^ ^]^ The design of partially charged polymer networks can be guided by two parameters (Figure 1) that reflect these interactions: 1) The integral volume charge density, defined as the total number of charged moieties (sulfate groups) in the swollen hydrogel network (P1 in µmol mL^−1^), and 2) the local charge pattern along a single GAG chain, defined as the number of charged moieties (sulfate groups) per molecular weight of the GAG repeating unit (P2 in mmol per g mol^−1^). Positively charged proteins, in particular chemokines, are primarily attracted by the integral negative charge of the starPEG‐GAG‐based hydrogels as characterized by P1 (Figure 1A), a parameter that is influenced by intermolecular interactions of GAG chains within the polymer network. If the protein further approaches a gel‐contained GAG chain, condensed counter‐ions become released, resulting in a gain of entropy—considered to be the main driving force for binding (Figure 1B)—followed by the formation of spatially matching salt bridges between amino acids of the protein and sulfate moieties of the GAG chain (Figure 1C). The collective effect of P1 and P2 determines the interaction of any charged protein with starPEG‐GAG‐hydrogel networks. Crosslinking of heparin or selectively desulfated heparin derivatives (displaying different P2 values) with starPEG allows for independently tuning of both parameters to maximize the sequestration of proteins of interest.^[^ ^23^ ^]^ Accordingly, our related optimization of both parameters P1 and P2 aimed at maximized sequestration of pro‐inflammatory chemokines Interleukin‐8 (IL‐8, CXCL8) and monocyte chemoattractant protein 1 (MCP‐1, CCL2),^[^ ^15^ ^]^ which beyond other chemokines have been previously described to predominantly contribute to the sustained inflammation found in chronic wounds,^[^ ^11^, ^24^, ^25^ ^]^ without affecting the levels of pro‐regenerative factors of the wound milieu.

**Figure 2:**
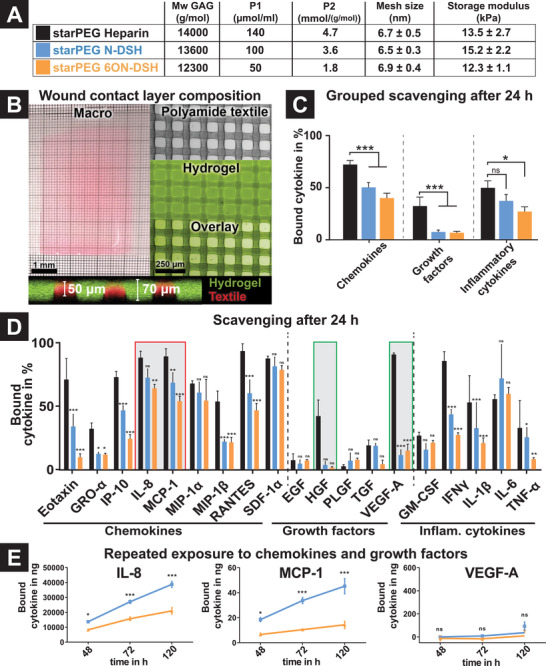
Characterization of the starPEG‐GAG hydrogel‐based WCL composite dressings. A) Physicochemical properties of the different starPEG‐GAG hydrogels: *M*_w_ GAG: molecular weight of the GAG building block, P1: global charge density of the hydrogel, P2: local charge density of the hydrogel, mesh size and storage moduli of the different hydrogel types. B) Structure of the resulting starPEG‐GAG hydrogel‐based WCL composite dressing (a representative example of an N‐DSH‐ hydrogel‐based WCL is shown). C) Summary binding characteristics of chemokines, growth factors, and inflammatory cytokines to the different starPEG‐GAG hydrogel‐based WCL composite dressings. D) Binding characteristics of the individual signaling mediators to the different starPEG‐GAG hydrogel‐based WCL composite dressings. Significance is indicated against the corresponding starPEG‐heparin hydrogel‐based WC composite dressings. Absolute concentrations can be found in Table S1, Supporting Information. E) Binding characteristics of starPEG‐N‐DSH‐ and ‐6O, N‐DSH‐based WCL composite dressings upon repeated exposure in simulated wound fluid (SWF). Bars represent calculated means ± SEM for *n* = 3 independent experiments. *p*‐values are calculated using one‐way ANOVA with Bonferroni correction, ns, not significant, *p* ≤ 0.05, ^**^ for *p* ≤ 0.01, and ^***^ for *p* ≤ 0.001.

Considering similarities of the chemokines to be sequestered—both IL‐8 and MCP‐1 display binding motives with up to five spatially arranged positively charged Lys and Arg residues and an overall high positive charge density^[^ ^14^ ^]^—and differences to the properties of wound‐healing related growth factors, such as hepatocyte growth factor (HFG), placental growth factor (PLGF), transforming growth factor (TGF), or vascular endothelial growth factor A (VEGF‐A), a range of P1 and P2 conditions were pre‐selected to guide our hydrogel design, based on the results of a recent screen.^[^ ^23^ ^]^ To meet the related requirements of the intended wound dressing application, we selected a) fully sulfated (therefore anticoagulant) heparin, b) *N*‐desulfated heparin (*N*‐DSH), and c) 6O, *N*‐desulfated heparin (6ON‐DSH) with 60% and 30% of the sulfate groups remaining as building blocks of starPEG‐GAG hydrogels, that have been crosslinked with amino‐end‐functionalized 4‐arm starPEG as previously described.^[^ ^15^, ^26^ ^]^ Selective desulfation and polymer network adjustments provided a twofold and sixfold decrease in the integral volume charge density P1 from 140 to 100 and 50 µmol mL^−1^ and a decreasing P2 from 4.7 to 3.6 and 1.8 mmol per g mol^−1^ when comparing hydrogels made of heparin, N‐DSH or 6ON‐DSH, respectively. Adapting the starPEG‐GAG‐hydrogel formation conditions by varying the crosslinking degree and overall solid content of the hydrogel precursor solutions allowed us to keep the mechanical properties (storage moduli ≈ 11–15 kPa) and the mesh size of the compared materials in a similar range of ≈6–7 nm (**Figure** **2**A). The mechanical properties of the starPEG‐GAG‐based hydrogels were chosen to ensure the integrity of the resulting WCL composite dressings during processing, handling, and application while allowing for fast and sterically unhindered transport of chemokines (with average radii of 3 nm) through the hydrogel network.^[^ ^14^, ^15^ ^]^

A fundamental requirement for a WCL composite dressing concerns the stability of the hydrogel against delamination and abrasion. We aimed at meeting these criteria by combining an interwoven textile with the above selected starPEG‐GAG hydrogels into a composite wound dressing system (Figure 2B): The textile provides mechanical support and enables wound exudate transport while the GAG‐based hydrogel modulates the inflammation as described above and contributes to the hydration and low adhesiveness of the WCL.^[^ ^15^ ^]^ To form the starPEG‐GAG‐hydrogel based WCL composite dressings, the hydrogel precursor solution was spread onto the textile mesh, which was subsequently kept between two ECTFE (ethylene chlorotrifluoroethylene)‐coated glass slides until crosslinking was completed. During this process, the hydrogel precursors penetrated the textile and formed a 70 µm thick coherent gel layer surrounding the textile fibers (Figure 2B). To ensure the reproducible preparation and stability of the starPEG‐GAG‐hydrogel based WCL composite dressings, the mixing and pH‐regime of the hydrogel precursors were carefully adjusted to control the crosslinking process of the hydrogel network. The resulting starPEG‐GAG‐hydrogel based WCL composite dressings were found to be stable against abrasion and delamination by mechanical testing with a repeated load of 100 mN (Figure S1, Supporting Information). In addition, increased tension resistance was observed for the starPEG‐GAG‐hydrogel based WCL composite dressings with 1.3 times higher breaking load compared to starPEG‐GAG‐based hydrogels without textile support (Figure S2, Supporting Information).

Next, the starPEG‐GAG‐hydrogel based WCL composite dressings were tested for their sequestration characteristics by 24 h incubation with a range of cytokines and growth factors involved in inflammation and wound repair. StarPEG‐GAG‐hydrogel based WCLs made of N‐DSH bound 50.4 ± 23.4% of all chemokines and a slightly lower amount of other pro‐inflammatory cytokines (37.5 ± 23.7%), while 6ON‐DSH‐based WCL composite dressings sequestered a slightly lower quantity of both chemokines and cytokines (40.2 ± 24.0% for chemokines and 27.2 ± 18.0% for inflammatory cytokines, Figure 2C). Native heparin‐based WCL composite dressings bound more chemokines and inflammatory cytokines (72.3 ± 20.0% and 49.9 ± 25.2%) but also depleted more than five times higher amounts of the pro‐regenerative growth factors (heparin: 32.5 ± 33.2, N‐DSH: 7.8 ± 6.8, and 6ON‐DSH: 7.0 ± 5.2). However, the pro‐inflammatory chemokines (IL‐8, MCP‐1, MIP‐1a, RANTES, and SDF‐1*α*) were found to be sequestered by N‐DSH and 6ON‐DSH based WCL composite dressings to 70–90%, that is, nearly as efficiently as by the respective heparin‐based WCL composite dressing (Figure 2D). IL‐8, a neutrophil chemoattractant, and MCP‐1, a monocyte chemoattractant, in particular, are inflammatory key mediators of chronic wounds and correlate with the prolonged presence of immune cells in the wound tissue.^[^ ^24^, ^27^ ^]^

As a next step toward the application, the long‐time chemokine scavenging performance of the developed WCL composite dressings was analyzed upon repeated exposure to a simulated wound fluid (SWF), a solution that closely mimics the chemokine and growth factor levels, salinity, and albumin concentration of human wound exudates.^[^ ^15^, ^28^, ^29^ ^]^ While WCL composite dressings with hydrogels made of N‐DSH and 6ON‐DSH bound IL‐8 and MCP‐1, no binding of VEGF‐A was detected (Figure 2E). Moreover, repeated exposure to SWF revealed that WCL composite dressings based on N‐DSH as hydrogel building block displaying an integral volume charge density (P1) of 100 µmol mL^−1^ sequestered significantly more IL‐8 and MCP‐1 than WCL composite dressings based on 6ON‐DSH as hydrogel building block with P1 values of only 50 µmol mL^−1^ (Figure 2E). The more effective sequestration of the positively charged chemokines by N‐DSH based WCL composite dressings suggests a stronger contribution of unspecific binding effects due to electrostatic interactions (P1). In contrast, for the less positively charged growth factors, specific interactions seem to be more relevant for binding: Reducing P2 by selective desulfation of the GAG results in a lower binding of the pro‐regenerative factors.

Thus, our biologically inspired design approach of GAG‐derivate‐based biohybrid hydrogels, together with application‐related sequestration experiments using SWF, allowed us to identify the N‐DSH based WCL composite dressing to be superior for the intended application. Of note, the developed WCLs are easily adjustable to the shape and size of individual wounds, thereby facilitating regular dressing changes, and allow for optimal exudate management by a secondary dressing layer chosen according to the patient's specific wound characteristics.^[^ ^30^ ^]^

### 2.2 Assessing the Potential of Chemokine‐Scavenging Composite WCLs to Support the Healing of Chronic Cutaneous Wounds in a Porcine Model

N‐DSH and 6ON‐DSH hydrogel‐based WCL composite dressings were chosen due to their superior scavenging selectivity, graded chemokine sequestration performance and absent anticoagulant activity^[^ ^31^, ^32^ ^]^ for in vivo testing in a porcine model of chronic cutaneous wounds. This model has been previously described to be excellent to study delayed wound healing as the porcine tissue shows substantial similarities to the human skin physiology, similar healing response, and high concordance with human study outcomes.^[^ ^33^, ^34^, ^35^, ^36^ ^]^ The onset of diabetes mellitus was induced by streptozotocin injection (100 mg kg^−1^) two weeks before surgery and confirmed by the increase of the blood glucose levels (Figure S3, Supporting Information). While this model resembles more an acute than a chronic diabetic state, it shows a significant delay of reepithelialization as found in chronic wounds of human patients.^[^ ^36^ ^]^

**Figure 3:**
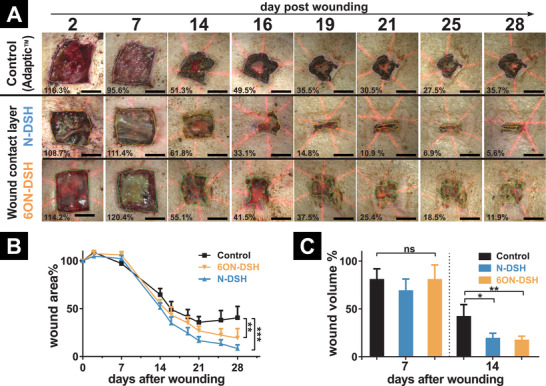
Characterization of wound healing of chronic wounds. The wounds were treated with starPEG‐N‐DSH or ‐6O, N‐DSH hydrogel‐based WCL composite dressings, or the standard of care (Adaptic) WCL control dressing. A) Representative images (*n* = 1) of wound healing over 28 days. Relative wound area is indicated in the bottom left of every image. Scale bars = 1 cm. B) Mean wound area over time. C) Mean wound volume at 7 and 14 days post wounding. For (B) and (C) *n* = 6 for control wounds and *n* = 12 for each set of starPEG‐GAG hydrogel‐based WCL composite dressing treated wounds until day 21 and *n* = 3 for control wounds and *n* = 6 for each set of starPEG‐GAG hydrogel‐based WCL composite dressing treated wounds until day 28 were analyzed (see Table S3, Supporting Information). *p*‐values are calculated using one‐way ANOVA with Bonferroni correction, ns, not significant, *p* ≤ 0.05, ^**^ for *p* ≤ 0.01, and ^***^ for *p* ≤ 0.001.

The 2 × 2 cm full‐thickness excision wounds were treated with either one of the two starPEG‐GAG‐hydrogel based WCL composite dressings or, for the control group, with a commercially available non‐adhesive WCL (Adaptic), which is widely used for low exudate wounds as found in the porcine wound model and previously shown to promote reepithelialization.^[^ ^37^, ^38^, ^39^ ^]^ Wounds in the treatment and control group were covered with an adhesive, semi‐occlusive film dressing (Hydrofilm) on top of the primary dressing. While a range of functional dressings is clinically applied in the treatment of chronic wounds to match their respective protease and exudate levels, we selected one widely used dressing as a control that best represents the non‐pharmacological support of various wounds by protection and moisture management. After the first 14 days, an increased wound area reduction rate could be observed in the wounds treated with either type of starPEG‐GAG hydrogel‐based WCL composite dressing (**Figure** **3**A) compared to the Adaptic WCL treated control wounds. Both starPEG‐GAG hydrogel‐based WCL composite dressings supported nearly full wound closure in all treated animals 28 days post wounding. The low adhesiveness of the starPEG‐GAG hydrogel‐based WCL composite dressings was evident from the absence of any tissue adhesion to the composites when they were removed from the wounds, which might be caused by the high amount of bound water and low protein affinity of the PEG‐building block.

While N‐DSH and 6ON‐DSH hydrogel‐based WCL composite dressings treated wounds showed a significant decrease in wound area over 28 days to 8.3 ± 4.0% (*n* = 6) and 19.3 ± 10.0% (*n* = 6) respectively, the control wounds with the standard of care dressings remained at 40 ± 11.7% (*n* = 3) of the initial wound area with a delayed reepithelialization of the defect site (Figure 3B). Through 3D tissue scanning, the wound volume was assessed during the healing process. While no significant differences could be found seven days post wounding, a twofold lower wound volume for the treatment with both starPEG‐GAG hydrogel‐based WCL composite dressings was identified at day 14 (Figure 3C). Of note, no further progression of wound healing could be observed in the control wounds after day 21 post wounding until the end of the study on day 28 (Figure 3B), whereas the wounds treated with either of the two starPEG‐GAG hydrogel‐based WCL composite dressings showed continuous healing.

The decrease of the wound area and volume demonstrate the positive effects of the starPEG‐GAG hydrogel‐based WCL composite dressings on the chronic wounds emerging from the depletion of chemokines from the wound bed and the resulting resolution of inflammation. In contrast, the standard of care dressing provides a beneficial wound environment but does not act on the inflammatory burden in the wound. While there were differences in the chemokine binding capacity between the hydrogels based on N‐DSH and 6ON‐DSH, no significant difference in the healing outcome of the respective WCL composite dressings could be detected. However, the stronger sequestration of IL‐8 and MCP‐1 by the starPEG‐N‐DSH‐hydrogel based WCL composite dressing might explain the trend observed in the slightly better healing performance of this WCL (wound area in Figure 3B), although these differences were not significant due to the high variability of the animal model. Due to the high GAG content of the hydrogels, the maximal binding capacity for chemokines is estimated at about 200 µg protein per cm^2^ of the WCL composite dressing. Considering typical levels of chemokines in wound exudates of chronic wounds being in the lower ng range per cm^2^ wound area (calculation see Table S2, Supporting Information), the capacity of both types of starPEG‐GAG hydrogel‐based WCL composite dressings is not exhausted within the application time. To further investigate the effects of the starPEG‐GAG‐based WCL composite dressings on the healing process, the wound tissue was collected and analyzed for proliferative and immunological characteristics.

### 2.3 Evaluating the Effect of GAG‐Functionalized WCLs by Histological Key Regeneration Parameters

**Figure 4:**
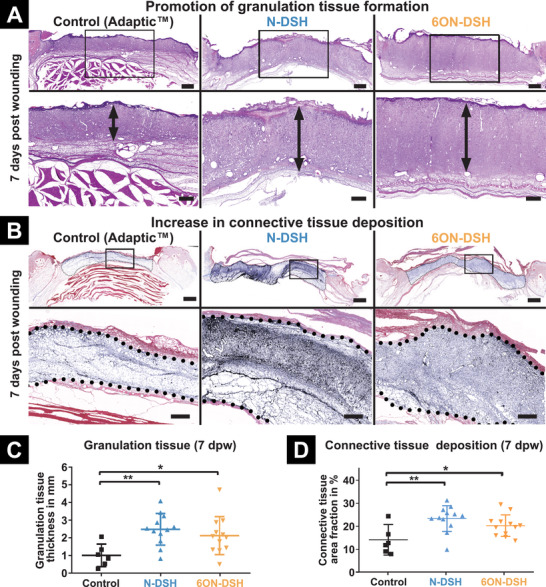
Histological analysis of newly formed tissue after seven days. A) Representative images of hematoxylin and eosin (H&E) stains of the wound tissue. Thickness of granulation tissue is indicated by a vertical, black arrow. Zoom is indicated by black rectangles. Scale bars represent 2000 µm in total wound images and 1000 µm in the magnified images. B) Representative images of Masson's Trichrome stains of the wound tissue. Color deconvoluted connective tissue (collagen fibers) within the granulation tissue is marked by the dotted outline. Zoom is indicated by black rectangles. Scale bars represent 2000 µm in total wound images and 500 µm in the magnified images. C) Quantification of the granulation tissue thickness. D) Quantification of the connective tissue deposition as area fraction. Each data point represents a single wound. For (C) and (D) *n* = 6 for control wounds and *n* = 12 for each set of starPEG‐GAG hydrogel‐based WCL composite dressing treated wounds have been analyzed. Values are given as mean with standard deviation. *p*‐values are calculated using one‐way ANOVA with Bonferroni correction, *p* ≤ 0.05, ^**^ for *p* ≤ 0.01, and ^***^ for *p* ≤ 0.001.

The formation of granulation tissue and the subsequent deposition of the newly formed ECM are critical events in the wound healing process. Fibroblasts migrating from the adjacent tissue enter the site of injury to proliferate and then secrete a collagen‐rich matrix, which builds the basis for angiogenesis and maturation of the newly formed tissue.^[^ ^40^, ^41^ ^]^ Median cross sections of the wound tissue seven days post wounding were analyzed to quantify granulation tissue formation and connective tissue density to identify the impact of the treatment with starPEG‐GAG hydrogel‐based WCL composite dressings on the granulation and ECM production (**Figure** **4**A,B).

While the formation of a granulation layer could be observed in all wounds, the tissue thickness was more than twofold increased in the starPEG‐GAG hydrogel‐based WCL composite dressing treatment groups (N‐DSH: 2.47 ± 0.90 mm (*n* = 12), 6ON‐DSH: 2.11 ± 1.08 mm (*n* = 12)) compared to the Adaptic WCL treated control group (0.99 ± 0.64 mm, (*n* = 6; Figure 4C)). Similarly, the amount of newly formed ECM was significantly enhanced in the wounds treated with the starPEG‐GAG hydrogel‐based WCL composite dressings indicated by the increased amount and density of connective tissue (mostly collagen fibers) observed in the newly formed tissue. In detail, starPEG‐GAG hydrogel‐based WCL composite dressing‐treated wounds showed an increased collagen area fraction of 23.30 ± 5.56% (*n* = 12) and 20.24 ± 4.65% (*n* = 12) for the N‐DSH‐ and 6ON‐DSH‐based WCLs compared to 13.82 ± 5.95% (*n* = 6) (Figure 4D) in the Adaptic WCL treated control group. The significant promotion of granulation tissue formation and matrix deposition indicates that these wounds have already progressed to a later stage of wound healing and thus demonstrates the potential of the developed starPEG‐GAG hydrogel‐based WCL composite dressings to promote the complete healing of chronic wounds. The effect of chemokine depletion on the increased granulation and ECM production might further be explained by a reduced tissue degradation rate through lower protease activity resulting from the resolved inflammation. Due to the sustained inflammation, an increase of matrix metalloproteases (MMP) and other less specific proteases can be commonly found in chronic wounds.^[^ ^42^, ^43^ ^]^ While these enzymes play an essential role in wound remodeling processes, such as debris removal, angiogenesis, and reepithelialization under acute healing conditions, their overexpression can degrade structural ECM components and growth factors, thus being a major factor for the delay in tissue repair.^[^ ^44^ ^]^

**Figure 5:**
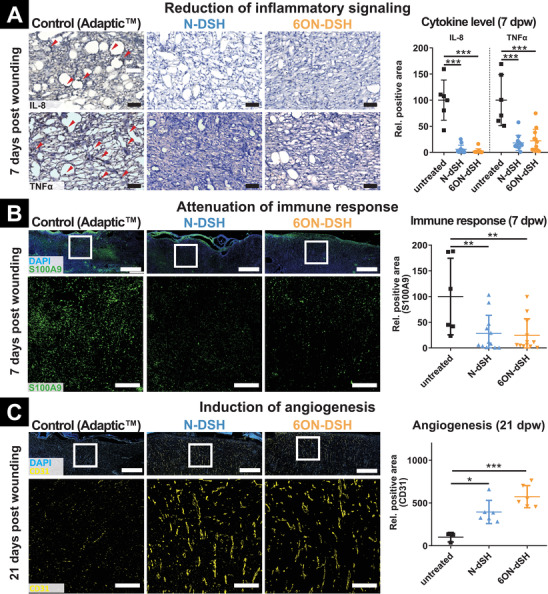
Immunohistochemical analysis of inflammation, immune cell invasion, and angiogenesis. A) Inflammatory cytokines (IL‐8 and TNF*α*) in the wound tissue seven days post wounding. Scale bars = 250 µm. Quantification of the positively stained area relative to control. B) Inflammatory S100A9+ neutrophils and macrophages in the wound tissue 7 days post wounding. Scale bars = 1000 µm in total wound images and 250 µm in the magnified images. Quantification of the S100A9+ area in the wound tissue relative to control. C) CD31+ vessels in the wound tissue 21 days post wounding. Scale bars = 1000 µm in total wound images and 250 µm in the magnified images. Quantification of the CD31+ area in the wound tissue relative to control. All values are given as mean with standard deviation. Each data point represents a single wound. For 7 days post wounding *n* = 6 for control wounds and *n* = 12 for each set of starPEG‐GAG hydrogel‐based WCL composite dressing treated wounds, and for 21 days post wounding *n* = 3 for control wounds and *n* = 6 for each set of starPEG‐GAG hydrogel‐based WCL composite dressing treated wounds were analyzed. *p*‐values are calculated using one‐way ANOVA with Bonferroni correction, *p* ≤ 0.05, ^**^ for *p* ≤ 0.01, and ^***^ for *p* ≤ 0.001.

Scavenging of chemokines from the wound bed can regulate the overall MMP activity in two ways: first by decreasing the chemokine levels, which have been demonstrated to induce MMP expression^[^ ^45^, ^46^, ^47^ ^]^ and second by reducing the overall amount of immune cells in the wound, as previously demonstrated, which are a leading source of MMP production in the wound environment.^[^ ^44^ ^]^ The decreased inflammatory state and reduced proteolytic environment of the wound allow for the promotion of healing and tissue repair response (**Figure** **5**).

Chemoattractant proteins, such as MCP‐1 and IL‐8, have been demonstrated to guide immune cell invasion from circulation and the surrounding tissue to the injury.^[^ ^11^ ^]^ To further study the attenuating effects of starPEG‐GAG hydrogel‐based WCL composite dressings on the inflammation state of the wound, we analyzed the prevalence of inflammatory cytokines tumor necrosis factor α (TNF*α)*and IL‐8 (Figure 5A) as well as the presence of inflammatory immune cells in the wound tissue (Figure 5B). Wounds treated with starPEG‐GAG hydrogel‐based WCL composite dressings showed a significant decrease of the key inflammatory regulator TNF*α* (N‐DSH: 18.1 ± 14.4% (*n* = 12), 6ON‐DSH: 22.0± 22.1% (*n* = 12) relative to the control wound (*n* = 6); Figure 5A) and the chemokine IL‐8 (N‐DSH: 5.825 ± 7.9% (*n* = 12), 6ON‐DSH: 2.4 ± 4.4% (*n* = 12) relative to the control wound (*n* = 6); Figure 5A).

Similarly when quantifying the residence of inflammatory neutrophils and macrophages characterized by S100A9 expression^[^ ^48^, ^49^ ^]^ at seven days post wounding, we found elevated cell densities in the Adaptic WCL treated control wound tissue, while both wounds treated with starPEG‐GAG hydrogel‐based WCL composite dressings showed significantly decreased cell counts (N‐DSH: 28.1 ± 35.3% (*n* = 12), 6ON‐DSH: 24.5 ± 32.1% (*n* = 12) relative to the control wound (*n* = 6); Figure 5B). Sequestration of chemoattractant mediators (IL‐8 and MCP‐1) by both starPEG‐N‐DSH and ‐6ON‐DSH hydrogel‐based WCL composite dressings resulted in a lower infiltration of the wound by inflammatory immune cells (Figure 5B).

Lastly, we analyzed the neo‐angiogenesis in the newly formed tissue after 21 days of healing. Chronic wounds impacted by insufficient angiogenesis have been described to show decreased vascularity and capillary density.^[^ ^50^ ^]^ The inadequate local angiogenesis is considered to contribute to the impaired healing in diabetic wounds.^[^ ^51^ ^]^ Because of the lack of vascularization, the wound remains hypoxic and undersupplied with nutrients essential for the formation of new tissue.^[^ ^52^ ^]^ When treated with the starPEG‐GAG hydrogel‐based WCL composite dressings, an increase of angiogenesis could be achieved (Figure 5C). After 28 days, the number of CD31^+^ vessels in the wound area had doubled in the wounds treated with starPEG‐GAG hydrogel‐based WCL composite dressings (N‐DSH: 394.3 ± 136.5%, 6ON‐DSH: 573.5 ± 129.2%) relative to the control wounds (Figure 5C). While previous studies successfully utilized hydrogels to deliver VEGF as a pro‐angiogenic signaling cue in wound healing,^[^ ^31^, ^53^ ^]^ no cytokine functionalization was required to increase angiogenesis within the tissue.

Thus, solely the decrease of the inflammatory burden in the wound allowed for faster healing, improved ECM deposition, and increased vessel formation to sustain the newly formed tissue.

## 3 Conclusion

Chronic wounds are characterized by a misbalance between inflammatory and regenerative signals: Augmented levels of chemokines in the wound tissue and the resulting increased infiltration by immune cells prolong the inflammation stage and impede healing.^[^ ^11^, ^25^ ^]^ Selective binding of inflammatory chemokines by starPEG‐GAG hydrogel‐based WCL composite dressings can modulate the wound signaling environment to dampen inflammation and attain a more pro‐regenerative state. Using a biologically inspired material design concept allowed us to tune the sequestration profiles of starPEG‐GAG hydrogels containing different selectively desulfated heparin derivatives. Adjusting both the integral and localized specific electrostatic interactions between proteins and biohybrid polymer hydrogel networks (characterized by their P1 and P2 charge density parameters) resulted in a maximized removal of pro‐inflammatory chemokines while keeping the levels of pro‐regenerative factors unaffected. Non‐adhesive and biostable textile composite WCLs containing these hydrogels were engineered to provide mechanical integrity without affecting the exudate management functionality of respective top dressings. This may allow using the starPEG‐GAG hydrogel‐based WCL composite dressings as inflammation resolving elements in various different well‐established wound treatment systems.

As a proof of concept, inflammation in a porcine chronic wound model treated with the starPEG‐N‐DSH‐ or 6ON‐DSH hydrogel‐based WCL composite dressing was quickly resolved, enabling the formation of vascularized tissue and a complete wound closure within 28 days. Our reported data show in a large animal model of human wound healing disorders that resolving the excessive inflammatory milieu of chronic wounds by starPEG‐GAG hydrogel‐based WCL composite dressings can rescue healing and functional tissue repair. While significant differences between N‐DSH‐ and 6ON‐DSH hydrogel‐based WCL composite dressings occur for repeated chemokine sequestration in vitro, both WCLs performed similarly well with respect to wound healing, granulation tissue formation, connective tissue deposition, immune response, and angiogenesis in vivo.

The clinical translation of our therapeutic starPEG‐GAG hydrogel‐based WCL composite dressings will be facilitated by their stability in the wound healing context and the absence of any bioactive moieties. Beyond dermal wound healing, similar chemokine scavenging technologies using starPEG‐GAG‐based biohybrid polymer hydrogels are envisioned to create powerful new opportunities for therapeutic intervention in other diseases caused by or associated with excessive inflammation such as hypercytokinemia (“cytokine storm”), arthritis, inflammatory bowel disease, and several others.

## 4 Experimental Section

### Preparation of StarPEG‐GAG Hydrogel‐Functionalized WCLs

StarPEG‐GAG hydrogels with an approximate thickness of 70 µm were prepared on top of interwoven textile materials by spreading the unpolymerized hydrogel mixture onto the textile between two hydrophobic glass plates. The assembling of the starPEG‐GAG hydrogels, as well as the synthesis of the desulfated heparin derivatives, were performed and analyzed as previously described.^[^ ^26^, ^54^, ^55^, ^56^ ^]^ In brief, in‐house synthesized N‐DSH and 6ON‐DSH, heparin (Sigma Aldrich), 1‐ethyl‐3‐(3‐dimethyl aminopropyl) carbodiimide (EDC, Sigma Aldrich), *N*‐hydroxysulfosuccinimide (sulpho‐NHS, Sigma Aldrich), and amine end‐functionalized four‐arm starPEG (*M*_w_ = 10 000, Jenkem) were dissolved in deionized, decarbonized water on ice. After mixing the desulfated heparin with EDC and sulpho‐NHS (2:1 ratio of EDC:sulpho‐NHS), the solution was incubated on ice for 15 min for N‐DSH or heparin and 45 min for 6ON‐DSH, the activated heparin solution was mixed with starPEG to form hydrogels. For visualization of the hydrogel, 1% ATTO 647 labeled heparin (Atto‐Tec) was integrated into the hydrogel by adding it to the unpolymerized hydrogel mixture.

### Chemokine Binding to StarPEG‐GAG Hydrogel‐Functionalized WCLs

Surface‐bound starPEG‐GAG hydrogels (*n* = 3) were placed in custom‐made incubation chambers that allowed only minimal interaction of the protein solution with an area not originating from the hydrogel. A mixture of human growth factors, chemokines, and other cytokines (Table S1, Supporting Information) was incubated with the hydrogels at room temperature at the indicated concentration in DMEM medium supplemented with 0.1% v/v bovine serum albumin (Sigma Aldrich). After 24 h, supernatants were frozen in liquid nitrogen and stored at −80 °C until quantification of the remaining cytokines by multiplex bead‐based immunoassay (Thermo). All cytokine concentrations were normalized against a control group without hydrogel to account for degradation over the incubation period.

In order to more closely emulate the wound environment, SWF based on previously investigated chronic wound exudate was prepared.^[^ ^15^, ^28^ ^]^ Briefly, the SWF was prepared containing 5% BSA, 2.5 mm CaCl2, 142 mm NaCl, and a selection of cytokines at physiological concentrations: 700 ng mL^−1^ IL‐8, 1 ng mL^−1^ MCP‐1, and 22.5 ng mL^−1^ VEGF‐A. 7 mm hydrogel‐coated WCL discs were incubated with the SWF. After 48 h, the supernatants were frozen in liquid nitrogen and stored at −80°, and the discs were incubated with fresh SWF. The repeated SWF exposure procedure was performed over one week, and protein content in frozen samples was quantified by multiplex bead‐based immunoassay (Thermo).

### Mechanical Testing of StarPEG‐GAG Hydrogel‐Functionalized WCLs

Storage moduli of the hydrogels were assessed by oscillating measurements on swollen gel disks carried out on a rotational rheometer (Ares LN2, TA Instruments) fitted with 25 mm parallel plate geometry. Frequency sweeps were carried out at 25 °C in a shear frequency range of 1–100 rad s^−1^ with the strain amplitude of 2%. Both storage and loss modulus were measured as a function of the shear frequency. To estimate the mesh size (*ξ*) of the gels the following equation was used based on the rubber elasticity theory.^[^ ^57^ ^]^

(1) $$\xi \;={\left(\frac{G^{\prime }N_{A}}{RT}\right)}^{-\frac{1}{3}}\;$$

To assess the hydrogel delamination and abrasion from the interwoven textile, the WCLs were fixated on a UST‐Universal Surface Tester (Innowep) and abraded with a rubber ball tool head at 100 mN. As a control, a diamond tool head was used at the same force. Testing of tension deformation and the maximum load was tested on a static material testing machines (ZwickRoell) with at 1 m min^−1^ with a 10 N measurement cell.

### Pig Model and Diabetes Induction

Animal experiments were carried out at MD Biosciences according to the Israeli national legislation on the use of animals for experimental purposes and OECD guidelines (Animal experiment permit: IL‐16‐11‐382). The study comprised twelve female Danish X Large White Crossbred pigs. Diabetes induction, creation of the wounds, wound treatment, and data collection were performed under general anesthesia with isoflurane inhalation (Piramal). Diabetes was induced using streptozotocin (100 mg kg^−1^; Sigma Aldrich) and verified by a blood glucose level of 250 mg dL^−1^ or higher.

### Creation of Full‐Thickness Skin Wounds and Treatment

The animal was anesthetized, and full‐thickness wounds (2 × 2 cm) were inflicted in six different locations on the pig's back 1–2 cm on the left and right side of the spinal column using aseptic techniques. In each animal one wound was treated with the standard of care Adaptic (Johnson and Johnson) while two wounds were treated with either N‐DSH hydrogel‐based or the 6ON‐DSH hydrogel‐based WCL composite dressings. The assignment of each treatment per wound was done randomly. (One wound was used for a different only locally acting treatment not being part of this study.)

Following the excision and placement of the control or hydrogel WCL composite dressings, the wounds were covered with a secondary dressing comprised of a transparent adhesive semipermeable film (Hydrofilm, Hartmann). For pain control, a single injection of 1 mg kg^−1^ body weight Buprenorphine as given immediately after surgery. Moreover, osmotic pumps (Alzet) with a release of 0.083 mg kg^−1^ body weight per 24 h of Buprenorphine were implanted subcutaneously in the unwounded dorsal region directly after the wound surgery.

### Study Endpoint and Tissue Collection

Termination of the study was done by injection of a lethal dose of pentobarbitone sodium (intraperitoneal, >100 mg kg^−1^ body weight) according to Table S3, Supporting Information. For histological analysis, cross‐sectional wound biopsies 2 cm wide were taken from the middle of the wounds after removal of the dressings, including unwounded skin at the sides and subcutaneous tissue at the bottom. Half of the biopsy was shock‐frozen in liquid nitrogen for later protein extraction. The other half was fixed in 4% paraformaldehyde in PBS (Sigma Aldrich), following embedding in paraffin and sectioning in the middle of the wound (4 µm sections).

### Wound Area Reduction Rate

The wounds were assessed using a SilhouetteStar camera (Aranz medical) to measure wound area and depth. Wound size was measured on days 2, 7, 14, 16, 19, 21, 25, and 28 and blindly quantified by tracing the wound perimeter. The healing rate was calculated using the following formula:

(2) $$\mathrm{Closure}\;\mathrm{rate}\;=\;\frac{\mathrm{area}\;\mathrm{at}\;\mathrm{wounding}\;\mathrm{day}-\mathrm{area}\;\mathrm{at}\;\mathrm{day}\;X\;}{\mathrm{area}\;\mathrm{on}\;\mathrm{wounding}\;\mathrm{day}}\times \;100$$

For the representative images of the wound healing progress over time the images for a single representative wound per treatment group were centered, rotated, and cropped using ImageJ.^[^ ^58^, ^59^ ^]^

### Immunohistochemistry

The tissue sections were stained using Opal (PerkinElmer) staining kits (PerkinElmer) according to manufactures instructions. In brief, the 5 µm thick tissue sections were washed twice in xylene (Sigma Aldrich) for 10 min and then gradually rehydrated. Microwave‐assited Epitope retrieval with AR9 buffer (PerkinElmer) was done before blocking for 10 min, followed by primary antibody incubation in TBST (Sigma Aldrich) for 2 h. Mouse anti‐S100A9 (1:100, Abcam, ab22506) was used to evaluate inflammatory neutrophil and macrophage presence. After washing three times in TBST, secondary antibodies were incubated for 10 min followed by three times more washing in TBST and a 10 min staining with the Opal 520. The procedure was then repeated with rabbit anti‐CD31 (1:50, Abcam, ab9498) and Opal 690 to evaluate angiogenesis in the skin tissue.

Nuclei were counterstained with DAPI (4′,6‐diamidino‐2‐phenylindole) (Thermo), and fluorescent images were acquired using an automated slide scanner an Axio Scan.Z1 with a Plan‐Apochromat 20×/0.8 M27 (Zeiss). For the analysis of immune cell invasion and angiogenesis, all fluorescent structures within the granulation tissue (between the epidermis and underlying muscle layer (panniculus carnosus)) were quantified and divided by the total area of the sample using HALO (Indica Labs).

For the staining of cytokines a rabbit anti‐TNF (1:100, Abcam, ab6671) and mouse anti‐IL‐8 (1:100, Bio‐Rad, MCA1660) antibodies) were used together with a Vectastain Elite ABC‐HRP Kit with ImmPact DAB Substrate and hematoxylin QS counterstain (all Vector Laboratories) according to manufacturer's instructions. For the quantification of cytokine presence in the sections color convolution and thresholding of positively stained tissue in ImageJ^[^ ^58^, ^59^ ^]^ was used.

### Analysis of Tissue Structure

The tissue sections were stained with hematoxylin and eosin to evaluate the overall tissue structure and Masson–Goldner trichrome stain to evaluate connective tissue. The stained sections were observed and documented using an automated slide scanner an Axio Scan.Z1 (Zeiss) with a Plan‐Apochromat 20×/0.8 M27 (Zeiss). To analyze the deposition of connective tissue (collagen), ImageJ^[^ ^58^, ^59^ ^]^ was used as described by Ruifrok et al.^[^ ^60^ ^]^ In brief, the granulation tissue between the epidermis and underlying muscle layer (panniculus carnosus) was selected for each wound and color deconvoluted. The blue staining was evaluated for the area percentage of blue fibers within the total area.

### Statistical Analysis

Quantitative data are expressed as mean ± standard error of the mean (SEM.) or mean ± standard error (SD). Statistical analyses were performed in Prism 8 (Graphpad) using one‐way analysis of variance (ANOVA) with multiple comparisons with Bonferroni post‐test if not described otherwise. Any two‐sided *p‐*value less than 0.05 was statistically significant (* for *p* ≤ 0.05, ** for *p* ≤ 0.01, *** for *p* ≤ 0.001).

Porcine wound healing experiments were carried out at MD Biosciences, Israel. All other experiments were performed at Leibniz‐Institut für Polymerforschung Dresden e.V., Dresden.

## Conflict of Interest

The authors declare no conflict of interest.

## Author Contributions

L.S., U.F., and C.W. conceived the project. L.S. designed the material characterization and vivo study. L.S. performed all the experiments. P.A. and L.S. carried out the theoretical calculations. L.S., U.F., and C.W. wrote the manuscript. P.A. made helpful suggestions. All authors discussed the results and commented on the manuscript. C.W. and U.F. are inventors on the patent WO2010060485A1 “Bioactive hydrogel” that covers the starPEG‐GAG hydrogels used in the study and L.S., P.A., C.W., and U.F. are inventors of the patent application PCT/DE2018/100218 that covers charge‐controlled sequestration of inflammatory mediators by use of the starPEG‐GAG hydrogels.

## Supporting information

Supporting Information

## Data Availability

Research data are not shared.
