# Supplementary material for: Chemokine‐Capturing Wound Contact Layer Rescues Dermal Healing
Source: Adv Sci (Weinh). 2021 Jul 18;8(18):2100293. doi: 10.1002/advs.202100293 (PMC8456214; doi:10.1002/advs.202100293)
Supplement: Supplementary file 1 — Supporting Information [file ADVS-8-2100293-s001.pdf]

WILEY-VCH

© 2021 Wiley-VCH GmbH

# Supporting Information

for *Adv. Sci.*, DOI: 10.1002/advs.202100293

# Chemokine-Capturing Wound Contact Layer Rescues Dermal Healing

*Lucas Schirmer, Passant Atallah, Uwe Freudenberg,* and *Carsten Werner**

# Supporting Information

## Chemokine-Capturing Wound Contact Layer Rescues Dermal Healing

*Lucas Schirmer, Passant Atallah, Uwe Freudenberg, Carsten Werner**

### Supplementary Materials

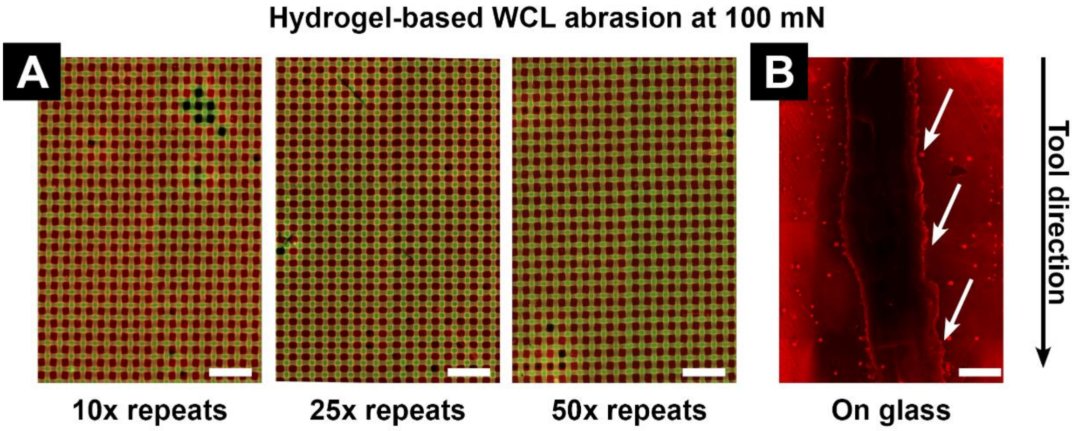


**Figure S1.** Abrasion testing of the starPEG-GAG hydrogel-based WCL composite dressing at 100 mN. (A) - Rubber hemisphere tool head was repeatedly wiped over the hydrogel-functionalized textile surface at 100 mN. (B) - For the control measurements on a hydrogel-coated glass surface showed instant hydrogel delamination as indicated by the white arrows.

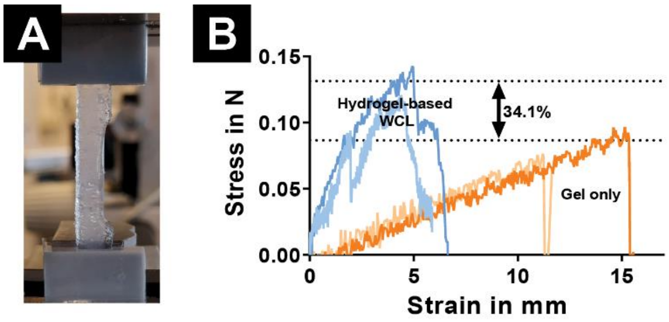


**Figure S2. Tensile tests of hydrogel and hydrogel-based WCL. (A)** - Tension test setup with a dog bone-shaped hydrogel. **(B)** - Results of tension analysis for starPEG-GAG hydrogels and the hydrogel-based WCL.

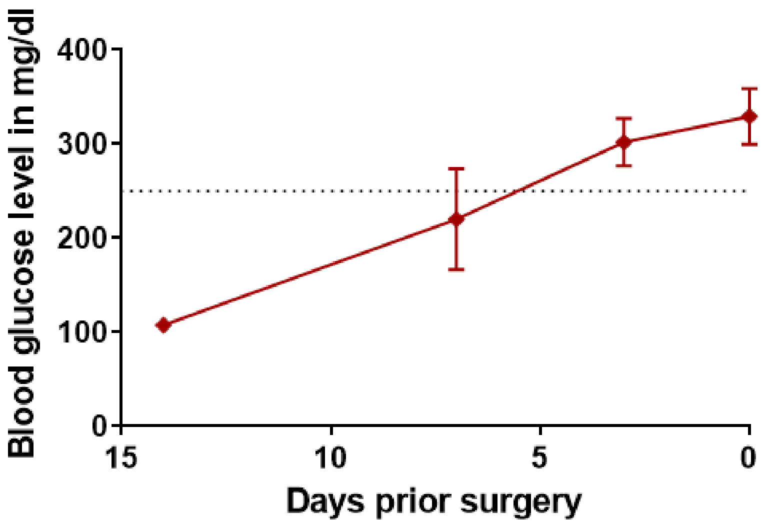


**Figure S3**. **Monitoring of porcine blood glucose levels after diabetes induction.** Increase in blood glucose level over 250 mg/dl (dashed line) before wounding. Values for n=12 animals are given as mean with standard deviation.

**Table S1. Concentrations of various signaling mediators used for scavenging assay.**

| Chemokines | |
|---|---|
| **Eotaxin** | 2 500 pg/ml |
| **GRO-alpha** | 9 500 pg/ml |
| **IL-8** | 8 900 pg/ml |
| **IP-10** | 8 800 pg/ml |
| **MCP-1** | 19 500 pg/ml |
| **MIP-1 alpha** | 6 400 pg/ml |
| **MIP-1 beta** | 13 500 pg/ml |
| **RANTES** | 3 600 pg/ml |
| **SDF-1 alpha** | 39 800 pg/ml |
| **Growth Factors** | |
| **EGF** | 9950 pg/ml |
| **HGF** | 30 600 pg/ml |
| **PLGF** | 6 800 pg/ml |
| **TGF** | 12500 000 pg/ml |
| **VEGF-A** | 23 500 pg/ml |
| **Cytokines** | |
| **GM-CSF** | 54 300 pg/ml |
| **IFN-gamma** | 30 600 pg/ml |
| **IL-1 beta** | 8 400 pg/ml |
| **IL-6** | 33 400 pg/ml |
| **TNF-alpha** | 26 700 pg/ml |

**Table S2. Estimated chemokine binding capacity for starPEG-GAG hydrogel-based WCLs.**

| | GAG component | | Chemokine (IL-8) |
|---|---|---|---|
| **Dressing type** | **Molecular weight** | **Amount** | **Estimated capacity for binding per cm$^2$ dressing (presuming one chemokine per three GAG molecules)** |
| Control | - | 0 mol/cm$^2$ | 0 µg |
| N-DSH dressing | 13600 g/mol | 6.7 x 10$^{-8}$ mol/cm$^2$ | 187.8 µg |
| 6ON-DSH dressing | 12320 g/mol | 6.8 x 10$^{-8}$ mol/cm$^2$ | 192.0 µg |

**Table S3. Wound samples collected per condition x days after wounding.**

| | **Day 7** | **Day 21** | **Day 28** |
|---|---|---|---|
| Control | 6 | 3 | 3 |
| N-DSH dressing | 12 | 6 | 6 |
| 6ON-DSH dressing | 12 | 6 | 6 |
